# Postpartum Exercise among Nigerian Women: Issues Relating to Exercise Performance and Self-Efficacy

**DOI:** 10.1155/2013/294518

**Published:** 2013-06-15

**Authors:** A. F. Adeniyi, O. O. Ogwumike, T. R. Bamikefa

**Affiliations:** Department of Physiotherapy, College of Medicine, University of Ibadan, Ibadan 200211, Nigeria

## Abstract

Physical exercise during postpartum period is beneficial to mothers, and the health gains are abundantly reported. This study characterises the postpartum exercise profile of a group of Nigerian women and reports how their exercise self-efficacies are influenced by sociodemographic characteristics. Participants were women attending the two largest postnatal clinics in Ibadan, south-western Nigeria. A self-developed questionnaire assessed the socio-demographic and exercise profile of participants, while the Exercise Self-Efficacy Scale assessed their exercise self-efficacy. About two-third (61.0%) of the participants were not aware that they could undertake physical exercise to enhance postpartum health, and 109 (47.8%) were not engaged in any exercise. Those who exercised did so for less than three days/week, and 89% of the women did not belong to any exercise support group. Exercise self-efficacy was significantly (*P* < 0.05) associated with being in an exercise programme, age, employment, work hours/week, monthly income, and number of pregnancies. Most of the women were not aware they could engage in postpartum exercise, and about half were not undertaking it. More women with high compared to moderate exercise self-efficacy undertook the exercise. Efforts at increasing awareness, improving exercise self-efficacy and adoption of postpartum exercise are desirable among the Nigerian women.

## 1. Introduction

Anatomical and physiological changes during pregnancy have the potential to affect the musculoskeletal, cardiovascular, and respiratory system [1]. Epidemiological studies also suggest that childbearing may contribute to the development of obesity [2]. Participation in a wide range of recreational activities appears to be safe during pregnancy in the absence of either medical or obstetric complications, and 30 minutes or more of moderate exercise a day on most, if not all, days of the week are recommended for pregnant women [3]. Women who exercise while pregnant have fewer complaints during pregnancy and have improved cardiovascular fitness and improved sense of self; and more than 90% of women who exercise during pregnancy continue to exercise after delivery [4].

Not only is exercise beneficial during pregnancy but even at the postpartum period which is the few weeks after delivery up to about 24 weeks. Generally, studies have established the importance of regular exercise during the postpartum period [2, 5]. Return to physical activity after pregnancy has been associated with decreased incidence of postpartum depression [1], anxiety and sleep disorders [4], prevention and treatment of urinary incontinence [4, 6], less likelihood to retain weight gained during pregnancy, and prevention of diastasis recti abdominus [4]. Postpartum exercise also improves aerobic fitness, high-density lipoprotein-cholesterol levels, insulin sensitivity, and psychological well-being [2]. Physical activity during postpartum is both a recommended and an essential contributor to maternal health [7].

The initiation and maintenance of regular exercise is generally a challenging and complex behavior. Pregnant women face unique challenges to exercise during pregnancy [8] and during the postpartum period [7]. However, studies have demonstrated that postpartum women are at high risk for physical inactivity and generally show lower levels of leisure-time physical activity compared to their prepregnancy state [9]. Understanding the beliefs, barriers, and enablers regarding physical activity during the postpartum period can help to more effectively tailor physical activity interventions [7]. Self-efficacy has particularly been shown to be important for overcoming pregnancy-related barriers [8], and it plays an important role as a mediator of exercise [10, 11]. Based on the principles of self-efficacy theory, intervention would be associated with improvements in perceptions of one's physical abilities to complete exercise (i.e., task self-efficacy), self-management competencies related to completing exercise (i.e., self-regulatory efficacy), satisfaction with one's body, overall negative mood, and actual physiological changes [12]. Although postpartum women have been shown to benefit maximally from physical exercise, before now, it is not known whether Nigerian women engage in postpartum exercise, and data on issues surrounding physical exercise of postpartum women from Nigeria was not available. This study was carried to (1) explore the physical exercise profile of a cross-section of postpartum women from Nigeria, (2) to characterize their exercise self-efficacy, and to (3) describe the link between sociodemographic factors and their exercise self-efficacy.

## 2. Materials and Methods

### 2.1. Participants

The participants in this study were consenting postpartum women who were attending postnatal clinics of the University College Hospital, Ibadan and Adeoyo Maternity Teaching Hospital, Yemetu, Ibadan. These two hospitals are the largest antenatal and postnatal clinics in Ibadan, south-western Nigeria. Participants who qualified for eligibility into the study were those who were not physically challenged and those who did not give history of being excluded from exercise participation on account of medical considerations. Using a purposive sampling technique, the eventual sample included in this cross-sectional study represents the total number of those who consented and who met the eligibility criteria within a 12-week data collection period.

### 2.2. Data Collection Instruments

A self-developed questionnaire was used to record the sociodemographic and exercise profile of the participants. Questions enquired about participants' age, marital status, religious affiliations, level of western educational attainment, employment status, and total monthly income. There were also questions on number of pregnancies and number of children. The participants were also asked whether they knew that they could engage in physical exercise within the postpartum period if there were no medical caveats. Questions also sought to know if any of them were already on any physical exercise, the type of exercise and whether they were registered or belonged to any exercise support group such as family exercise group, gymnasia group, swimming groups, or bicycling groups.

The Exercise Self-Efficacy Scale was used to retrieve data on the self-efficacy of the participants to undertake physical exercise. The scale measures the confidence in one's ability to persist with exercise in various situations representing the areas of negative affect, resisting relapse, and making time for exercise [13]. The scale is on a four-point Likert scale used to rate each item from 1 = not at all true to 4 = always true. The minimum score obtainable was 10, while the maximum was 40. A high score indicate a high level of self-efficacy, while a low score indicates a low level of self-efficacy. The Exercise Self-efficacy Scale had demonstrated reliability (Cronbach's alpha 0.917) and validity (correlated with minutes of exercise per week (*r* = 0.41; *P* = 0.0001) and health status (*r* = 0.37; *P* = 0.0001)) [14]. 

The questionnaire that assessed sociodemographic and exercise profile of the women and the Exercise Self-efficacy Scale were all translated from English to Yoruba language and the Exercise Self-efficacy Scale was taken through adaptability to the environment. The choice of Yoruba language was based on the fact that the study was conducted in the part of Nigeria that is predominantly occupied by Yoruba speaking people. The translation process involved the use of forward and backward translations by bilingual experts. The panel sat to identify and resolve any inadequate, vague, or ambiguous expressions and concepts. All identified discrepancies between the forward and backward translations and the existing versions of the questionnaires were also resolved and properly synthesised leading to the production of a complete translated version of the questionnaires. The final stage of the adaptation process was the pretest and was conducted on 20 postpartum women. Each participant completed the questionnaire and was interviewed to probe about what he or she thought was meant by each item on the questionnaires. All areas of difficulty encountered during the filling of the questionnaires were also identified and corrected.

### 2.3. The Data Collection Process

Ethical approval for the study was sought and obtained from the University of Ibadan/University College Hospital (UI/UCH) Health Research Ethics Committee (UI/EC/12/0114). Approval was also sought from the authorities of the two postnatal clinics in the hospitals where the study was carried out. A letter stating the purpose of the study, assuring the respondents of confidentiality, and seeking for their informed consent was distributed along with the questionnaires. The questionnaires were hand-distributed and collected by the researchers after completion. However, where immediate collection of the questionnaires was not possible, the collection was done on the next available clinic appointment.

### 2.4. Data Analysis

Descriptive statistics of frequencies and percentages were used to describe the data that were presented as categorical, while some other data were expressed as bar charts. The Exercise Self-efficacy Scale is presented in a continuum ranging from 10 as the lowest exercise self-efficacy and 40 as the highest. There were no cut-off points to describe that a participant has a low, moderate, or high exercise self-efficacies. Based on this, percentile grading was applied to the exercise self-efficacy data which was later converted to categories of low, moderate, or high exercise self efficacies. The exercise self-efficacy data were categorised into low, moderate, and high based on scores at the 25th, 50th, and 75th percentiles, respectively. In order to find association between exercise self-efficacy and each of participation in physical exercise and sociodemographic characteristics, the exercise self-efficacy scores were further categorised into those who had low to moderate exercise self-efficacy and those who had high exercise self-efficacy. Fisher's exact test was thereafter used to find association between exercise self-efficacy and each participation in physical exercise and sociodemographic characteristics. Statistical analyses were conducted using the International Business Machines (IBM) Statistical Package for the Social Sciences (SPSS) Version 20 (IBM Corporation, New York, NY, USA). Level of significance was set at *P* < 0.05.

## 3. Results

### 3.1. Sociodemographic Characteristics and Exercise Profile of the Participants

**Figure 1:**
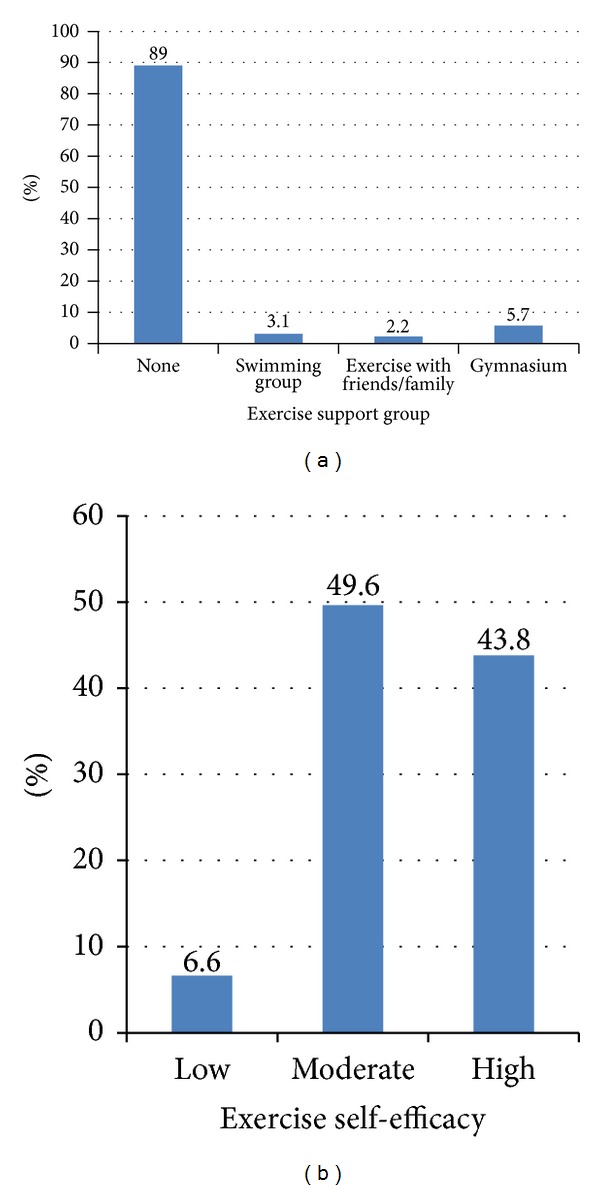
Exercise support group of respondents (a) and percentile scores in exercise self-efficacy (b).

The age ranges of most of the participants in this study were 21–30 years (45.6%) and 31–40 years (46.9%) with mean age of 31 ± 6.3 years (Table 1). More than half of them (52.2%) were self-employed, 140 (61.4%) reported a maximum of 48 hours of work per week, and majority of them (80.3%) gave a history of having one to three children. Other details of sociodemographic characteristics are presented in Table 1.  Table 2 shows that 139 (61.0%) of the women were not aware that they could undertake physical exercise to enhance health in the postpartum period, while close to half (47.8%) were not engaged in any physical exercise programme. However, for those who were engaged in physical exercise, brisk walking appeared to be the most adopted individual form of exercise (16.2%). Among those who were engaged in exercise programmes, 22 (18.5%) claimed to exercise only once in a week, while 20 (16.8%) claimed to undertake their mode of exercises daily. A total of 176 (77.2%) of the postpartum women reported that they had no any type of exercise enhancing gadget, but free weights were recorded as the most available and only among 20 (8.8%) of the participants. Majority of the women (89%) claimed they did not belong to any exercise support group (Figure 1(a)). The remaining one-tenth of the women was distributed into swimming, gymnasia, and family-facilitated exercise groups.

### 3.2. Exercise Self-Efficacy of Postpartum Women

The scores of exercise self-efficacy presented by the postpartum women are presented in Figure 1(b). The 25th percentile was marked by a score of 26 out of 40, while the 50th and 75th percentiles were marked by scores of 29 and 32, respectively. For the ease of interpretation, those who scored between 10 and 25 were classified as having low exercise self-efficacy, 26 to 28 as moderate, and 29 and over as high exercise self-efficacy. One hundred and thirteen (49.6%) of the participants presented with moderate exercise self-efficacy.  Table 3 presents the association between exercise self-efficacy and each of physical exercise participation and sociodemographic characteristics of the women. Out of the variables considered, it is observed that exercise self-efficacy was significantly associated with being in an exercise programme (*P* = 0.00001), age (*P* = 0.00001), employment status (*P* = 0.004), number of work hours per week (*P* = 0.00001), total monthly income (*P* = 0.00001), and number of pregnancies (*P* = 0.00002). The table shows that most of the women who were already in an exercise programme 101 (84.9%) reported high exercise self-efficacy compared to those who had moderate exercise self-efficacy where most of them (88.1%) reported no current exercise programme. The table also shows that significantly higher proportions of the women who were 30 years or younger (78%) had higher exercise self efficacies compared to those who were older than 30 years (20%). High exercise self-efficacy was also seen to be significantly more in those who were not employed (73.3%) compared to those who were in paid employment (46.5%). Although number of children did not significantly relate with higher exercise self-efficacy, a slightly higher proportion (50.3%) of those with lesser number of children were seen to have higher exercise self-efficacy compared to those with more than three children. 

## 4. Discussion

This study was carried out to explore the physical exercise profile of a cross-section of postpartum women from Nigeria, to characterize their exercise self-efficacy and to describe the link between exercise self-efficacy, and each of participation in physical exercise and sociodemographic characteristics. The main findings from this study include the following: (1) more than half of the postpartum women were not aware that they could engage in health enhancing physical exercise in the postpartum period; (2) close to half of them were not involved in any exercise programme, and majority of them did not belong to any exercise support group; (3) for those who were engaged in physical exercise, brisk walking was reported as the most adopted form of activity, and more than one-third of the women did the exercise for less than three days in a week; (4) about half of the women had moderate exercise self-efficacy; (5) being in an exercise programme was associated with exercise self-efficacy, and exercise self-efficacy was found to be linked with age, employment status, number of working hours per week, monthly income, and number of pregnancies. 

That most of the women in this study who were between the ages of 21 and 40 years may indicate the fact that this is the age-bearing range of the women in this study. This finding cannot be interpreted beyond this point because this might as well be by chance since the participants in this study were a sample of postpartum women from only two hospitals in Ibadan. This might not be representative of the entire postpartum women in Ibadan who may have registered for their pre- and postnatal care in many other government and private hospitals within and around the city. In a previous study that considered 349 pregnant women in Nigeria, about two-third of the women were between 25 and 35 years [15], while in another study of 518 pregnant women, more that 80% were in the 20–39 years bracket [16]. Information was sought on the number of pregnancies and children that the women have had. It was observed that majority of them have had one to three pregnancies and one to three children. A previous study on household size and composition conducted in the south-western city of Abeokuta [17] which happens to be from the same geographical location with Ibadan where this present study was carried out also reported the highest number of children to be four (29.8%) followed by three (23.5%). 

For the overall health of women in the postpartum period, physical exercise has been well documented to be necessary and beneficial [2, 4, 5]. Scott [4] also reported that most women actually want to exercise after childbirth to lose the weight they gained during pregnancy. Notwithstanding the numerous documentations on these benefits, more than half of the women in this present study reported that they were not aware that they could engage in health enhancing physical exercise within the postpartum period. As a followup, it was discovered in this study that about half of the women were actually not engaged in any form of physical exercise during the postpartum period. This observation is against the report that women with no complications during pregnancy or delivery can resume exercise immediately after the delivery [3, 4]. It has also been reported that rapid resumption of exercise has no adverse effects, although gradual return to former activities is advised [18]. The lack of awareness exhibited by the women could, however, be an apparent contributor to the poor subscription to postpartum exercise seen among the women in this study.

Although many exercise options were being adopted by the women who were into exercise during the postpartum period, it was observed that brisk walking was the most subscribed individual type of exercise, while fairly more women reported combining two or more exercise modalities. The reason for adoption of brisk walking as the most applied individual exercise is not fully known, but it is taught that its simplicity and the fear of perceived risks associated with more demanding exercises may be the strong factors in favour of its adoption. It has been reported that some women are afraid to participate in physical activity, especially vigorous exercises, thinking it will negatively impact breast milk production and breastfeeding [4]. Whether the women did the brisk walking at health-enhancing intensity or not is not known because the speed at which they walked could not be ascertained. Brisk walking that is carried out at an energy requirement of 3–5 metabolic equivalents (METS) is equivalent to brisk walking at 3-4 mph, and this yields a moderate intensity activity level [18]. While the exercise types of the few women who engaged in exercise cut across activities like walking, swimming, cycling, jogging, and weight lifts, more than one-third of them were only doing it for less than three days in a week. This may not be sufficient for health gains as the Centers for Disease Control and Prevention and American College of Sports Medicine recommendation for exercise aimed at improving the health and well-being of nonpregnant individuals, suggest that an accumulation of 30 minutes or more of moderate exercise a day should occur on most, if not all, days of the week [18, 19]. 

The availability of exercise equipment such as bicycle ergometer, treadmill, and free weights among others, which may facilitate physical exercise among the women in this study, was highly limited as about three out of four could not report possessing any equipment. This might have played a role in the poor adoption of exercise among the women. For instance, some postpartum women had indicated that having home exercise equipment facilitated or would facilitate their physical activity [20]. Apart from the lack of personal exercise equipment, almost all the women were also not attached or registered with any exercise group or have a link with any family exercise group. Only a little fraction could claim registration with an exercise group or family exercise buddy. This might have also affected their adoption of exercise behaviour. According to Scott [4], women who are most successful at incorporating a regular routine of physical activity into their life after delivery have higher self-esteem and feel more supported by family and friends. The author further claimed that if women have support for exercise from family and friends, both in the form of verbal encouragement and companionship during exercise, they will more likely initiate and maintain a regular exercise program. This assertion has also been withheld recently by Nicklas et al. [20] who reported that finding an exercise buddy or a group (including group exercise classes) facilitated exercise participation among postpartum women. Gyms and community groups could facilitate physical activity for mothers of newborns by scheduling classes or group activities for the women together [4, 20].

Exercise self-efficacy has been shown to be an important regulator of exercise behaviour [8, 10–12]. It was interesting to note that close to half of the women in this study presented with moderate exercise self-efficacy, while another slightly lower proportion presented with high efficacy level. However, most of the women who reported that they were already engaged in exercise programmes were the ones with high, not moderate exercise self-efficacy. This implies that moderate amount of exercise self-efficacy, though numerically good, may not practically suffice to get the group of women in this study to exercise as more of those with high exercise self-efficacy were the ones observed to engage in exercise programmes. The reason for a prerequisite of high exercise self-efficacy is not known, but it is observed that being in exercise programme was significantly linked with higher exercise self-efficacy, and exercise self-efficacy was itself significantly linked with age, employment status, number of working hours per week, monthly income, and number of pregnancies. Some of these sociodemographic characteristics may have served as either facilitators or barriers to attainment of postpartum physical exercise. For instance, women with newborn babies list many barriers to physical exercise with the most common being lack of assistance with childcare and insufficient time [21, 22]. Another report on postpartum women with gestational diabetes documents barriers to physical activity to include lack of motivation/fatigue, lack of time, and financial constraints [20]. 

The potential clinical benefit of this study is that it has been able to shed more light on a number of issues surrounding the participation of a group of Nigerian women in postpartum physical exercise. With the findings from this study, it has become known that a group of postpartum women from Nigeria are not into postpartum exercises, and this will serve as documented evidence to the fact that the women are yet to maximally benefit from the various clinical health benefits associated with postpartum exercise. The array of details that have relevance with the promotion of physical exercise among these women that have been revealed in this study can also be looked into in the planning of realistic exercise programmes for women in this group. A striking observation in this study is the fact that being involved in postpartum exercise requires high rather than moderate exercise self-efficacy meaning that this psychological construct needs to be amplified in order to get this group of women to exercise. When individuals are able to improve perceptions of both their physical abilities (i.e., task self-efficacy) and ability to overcome barriers to exercise (i.e., self-regulatory efficacy), they tend to maintain their assigned exercise regimens better [12]. 

This study is not without shortcomings. The first one bothers on the relatively small sample size considering the fact that the women in this study were recruited from only the two largest hospitals in Ibadan. Hence, this sample may not necessarily represent the physical exercise profile of postpartum women from other hospital settings. In addition, it could not be ascertained whether the lack of exercise in a majority of the women were influenced by a number of other factors that were not considered in this study. Such factors include their prepregnancy exercise levels and personal motivations. It is, however, observed that the exercise self-efficacy which plays a major role in exercise participation is mediated by a number of sociodemographic characteristics that have been listed in this study. These characteristics may be looked into in an attempt to improve exercise self-efficacy of postpartum women.

It is concluded that most of the Nigerian postpartum women who participated in this study were not aware that they could engage in physical exercise during their postpartum period and hence most of them were not participating in the exercise. Most of the women had moderate to high level of exercise self-efficacy, but participation in postpartum physical exercise was linked to a high exercise self-efficacy. High exercise self-efficacy was also linked with lower age, not in employment, lower hours of work per week, higher income, and higher number of pregnancies. Strategies to improve exercise self-efficacy and increase awareness and adoption of postpartum exercise among this group of women are highly desirable. For further studies, we recommend similar studies covering larger samples.

## Figures and Tables

**Table 1:** Sociodemographic characteristics of participants.

Variable	Frequency	Percentage (%)
Age		
15–20	14	6.1
21–30	104	45.6
31–40	107	46.9
41–50	3	1.3
Religion		
Christianity	147	64.5
Islam	81	35.5
Marital status		
Married	224	98.3
Single	1	0.4
Divorced	3	1.3
Level of education		
Nil	3	1.3
Primary	9	4.0
Secondary	55	24.1
Tertiary	161	70.6
Employment status		
Nil	8	3.5
Employed with wages	79	34.7
Voluntary worker	3	1.3
Student	19	8.3
Self-employed	119	52.2
Hours of work (hours/week)		
0–48	140	61.4
49–96	87	38.2
145–168	1	0.4
Total monthly income (in Naira)		
None	6	2.6
Less than 50,000	148	64.9
50,000–99,000	51	22.4
100,000–149,000	12	5.3
150,000 and above	11	4.8
Number of pregnancies		
1–3	196	86.0
4–6	31	13.6
7 and above	1	0.4
Number of children		
1–3	183	80.3
4–6	43	18.9
7 and above	2	0.8

**Table 2:** Exercise profile of the postpartum women.

Variable	Frequency	Percentage (%)
Aware of postnatal exercise		
Yes	89	39.0
No	139	61.0
Current exercise programme		
None	109	47.8
Brisk walking	37	16.2
Swimming	2	0.9
Cycling	1	0.4
Climbing stairs	19	8.3
Jogging	12	5.3
Weight lifts	2	0.9
Combinations	46	20.2
Usual number of days of exercise (*n* = 119)		
1 day	22	18.5
2 days	23	19.3
3 days	31	26.1
4 days	10	8.4
5 days	9	7.6
6 days	4	3.4
7 days	20	16.8
Exercise equipment		
None	176	77.2
Free weights	20	8.8
Balance ball	2	0.9
Treadmill	6	2.6
Stationary bike	8	3.5
Combination	16	7.0

**Table 3:** Association between socio-demographic characteristics and exercise self efficacy of the postpartum women.

Variable	*N*	Exercise self efficacy scores		*P*-value
		Low to moderate	High	
Current exercise programme				
Yes	119	18 (15.1)	101 (84.9)	0.00001*
No	109	96 (88.1)	13 (11.9)	
Age				
≤30	118	26 (22.0)	92 (78.0)	0.00001*
>30	110	88 (80.0)	22 (20.0)	
Religion				
Christianity	147	70 (47.6)	77 (52.4)	0.2032
Islam	81	44 (54.3)	37 (45.7)	
Marital status				
Married	224	113 (50.4)	111 (49.6)	0.3108
Not married	4	1 (25.0)	3 (75.0)	
Level of education				
Not educated	3	0 (0.0)	3 (100.0)	0.1233
Educated	225	114 (50.7)	111 (49.3)	
Employment status				
Not employed	30	8 (26.7)	22 (73.3)	0.004*
Employed with wages	198	106 (53.5)	92 (46.5)	
Hours of work (hours/week)				
≤48	140	37 (26.4)	103 (73.6)	0.00001*
49 and above	88	77 (87.5)	11 (12.5)	
Total monthly income in Naira				
Less than 50,000	154	108 (70.1)	46 (29.9)	0.00001*
≥50,000–99,000	74	6 (8.1)	68 (91.9)	
Number of pregnancies				
1–3	196	110 (56.1)	86 (43.9)	0.00002*
4 and above	32	4 (12.5)	28 (87.5)	
Number of children				
1–3	183	91 (49.7)	92 (50.3)	0.5
4 and above	45	23 (51.1)	22 (48.9)	

∗: significant.
